# Insight on Bacterial Newborn Meningitis Using a Neurovascular-Unit-on-a-Chip

**DOI:** 10.1128/spectrum.01233-23

**Published:** 2023-05-24

**Authors:** Rossana Rauti, Sharon Navok, Dvora Biran, Keshet Tadmor, Yael Leichtmann-Bardoogo, Eliora Z. Ron, Ben M. Maoz

**Affiliations:** a Department of Biomedical Engineering, Tel Aviv University, Tel Aviv, Israel; b Department of Biomolecular Sciences, University of Urbino Carlo Bo, Urbino, Italy; c The Shmunis School of Biomedicine and Cancer Research, Tel Aviv University, Tel Aviv, Israel; d Sagol School of Neuroscience, Tel Aviv University, Tel Aviv, Israel; e The Center for Nanoscience and Nanotechnology, Tel Aviv University, Tel Aviv, Israel; Weizmann Institute of Science

**Keywords:** bacterial meningitis, organ-on-a-chip, *E. coli*, neuronal network, vascular cells, neurovascular unit, electrophysiology, MEA, BBB permeability, *in vitro* models, newborn meningitis

## Abstract

Understanding the pathogenesis of bacterial infections is critical for combatting them. For some infections, animal models are inadequate and functional genomic studies are not possible. One example is bacterial meningitis, a life-threatening infection with high mortality and morbidity. Here, we used the newly developed, physiologically relevant, organ-on-a-chip platform integrating the endothelium with neurons, closely mimicking *in vivo* conditions. Using high-magnification microscopy, permeability measurements, electrophysiological recordings, and immunofluorescence staining, we studied the dynamic by which the pathogens cross the blood-brain barrier and damage the neurons. Our work opens up possibilities for performing large-scale screens with bacterial mutant libraries for identifying the virulence genes involved in meningitis and determining the role of these genes, including various capsule types, in the infection process. These data are essential for understanding and therapy of bacterial meningitis. Moreover, our system offers possibilities for the study of additional infections—bacterial, fungal, and viral.

**IMPORTANCE** The interactions of newborn meningitis (NBM) with the neurovascular unit are very complex and are hard to study. This work presents a new platform to study NBM in a system that enables monitoring of multicellular interactions and identifies processes that were not observed before.

## INTRODUCTION

The rapid development of antibiotic resistance resulted in an urgent need to find new avenues for combatting bacterial infections. The search for such avenues involves the identification of new targets for drug and vaccine development. To this end, it is essential to understand the pathophysiology of bacterial infections and the bacterial and host factors involved. One critical step for such understanding is the availability of a suitable model system in which it is possible to analyze the functional genomics of the pathogen and its host in a physiologically relevant system.

One severe bacterial infection is bacterial meningitis, a life-threatening infection of the central nervous system (CNS) with high morbidity and mortality. It is currently recognized as one of the top 10 killers in infection-related deaths worldwide, with almost half of the survivors suffering from diverse neurological sequelae (e.g., mental retardation, hearing impairment, and blindness) despite the advancements made in the field of antimicrobial treatment (1–4). The most common bacteria involved in meningitis were strains of Neisseria meningitidis, but these infections are drastically reduced by the recent development of meningococci vaccines and adjuvant therapies (5, 6). However, there are increasing types of meningitis caused by bacteria other than N. meningitidis, especially Gram-negative bacteria such as extraintestinal pathogenic Escherichia coli (ExPEC) (7, 8). These bacteria are usually involved in newborn meningitis (NBM) and are often antibiotic resistant (9). NBM affects up to 0.1% of all newborns, and chances of survival are about 50% in developing countries and between 8 and 12.5% in industrial countries, and those who recover are prone to developing neurodegenerative disease (10).

The study of bacterial meningitis is hindered by the difficulty of finding good model systems. The existing animal models are newborn mice, rats, or pigs (11–13). For example, our current knowledge about the pathogenic mechanisms that contribute to CNS complications and neuronal injury are largely derived from experimental models based on the direct injection of bacteria into the cerebrospinal fluid (CSF) and the bypassing of the blood-brain barrier (BBB) (2, 14–19). In these systems it is not possible to study the molecular and cellular mechanisms that are involved in the sequential steps of bacterium-host interactions or the downstream events that lead to cell death that is elicited by bacterial meningitis.

Another important factor in understanding bacterial meningitis is the identification of the virulence factors of the infecting bacteria. In order to understand which bacterial genes play a role in causing meningitis and how they are regulated, it is necessary to perform a genome-wide study using functional genomics and gene knockouts. Such experiments are most challenging in a model system based on newborn rodents.

During the past decades, *in vitro*-cultured brain microvascular endothelial cells (BMECs) have been developed to study the invading mechanisms of CNS-infecting pathogens through the BBB (20). In E. coli, several virulence factors associated with bacterial invasion of BMECs have been characterized (20–24). However, the BBB is more complicated than just a monolayer culture of endothelial cells, since there are multiple mechanical, biochemical conditions that are missing in the standard *in vitro* models, for example, the lack of flow that has significant effects on the BBB and the lack of interactions with the neurons and other cells. The most widely used *in vitro* BBB model is a two-dimensional (2D) Transwell system with an endothelial cell barrier on a porous membrane between the upper and lower chambers (25–27). This system, although useful for some studies, does not fully represent the complex *in vivo* structures and physiological functions of the human BBB and does not have neuronal components (28, 29).

**FIG 1:**
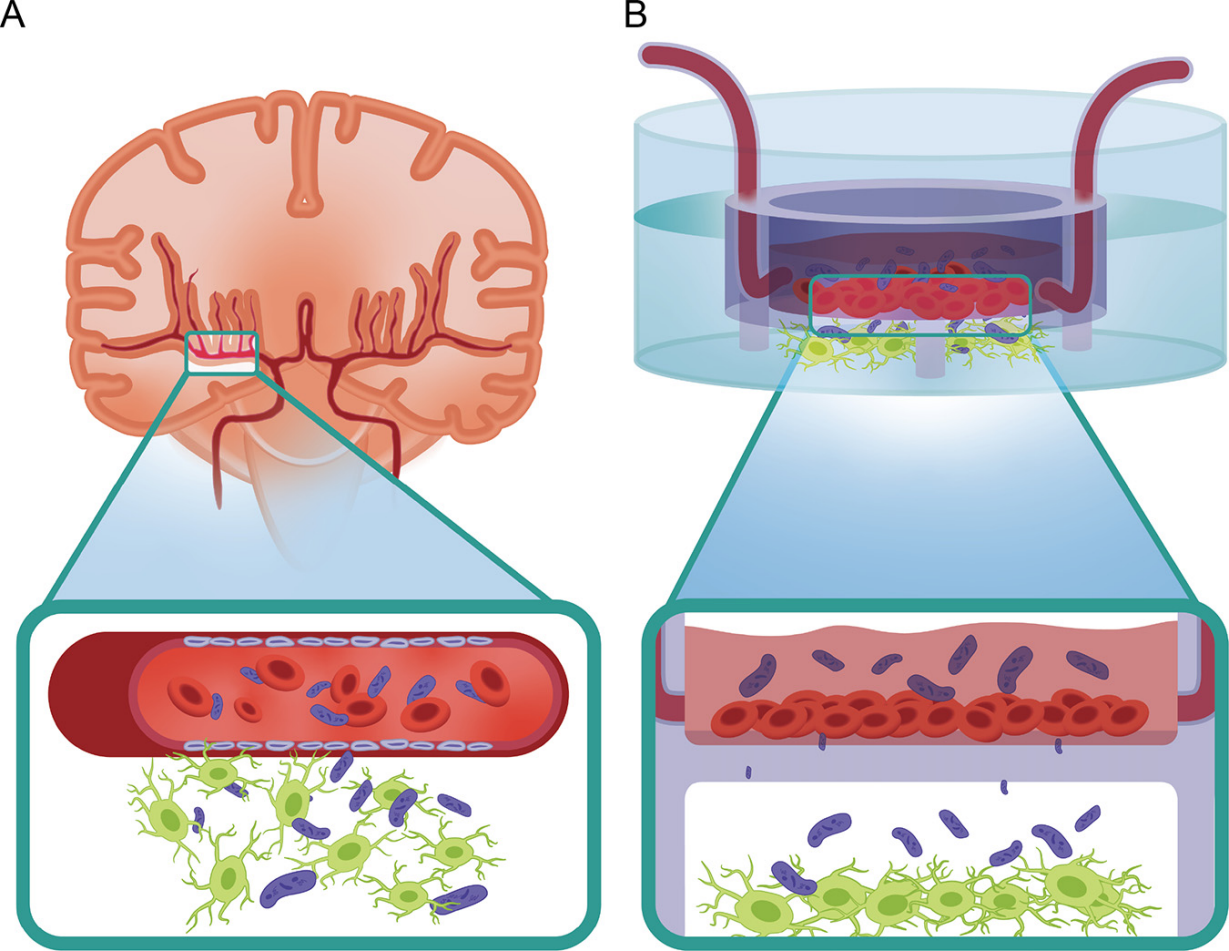
(A) Sketch representing the ability of meningitis E. coli bacteria to impair *in vivo* the BBB, with primary effect on the vasculature and then on the neurons. (B) Sketch of the platform used in our study: an insert chip able to mimic the BBB.

To overcome the limitations of the 2D-BBB model, organ-on-a-chip devices have been used to create *in vivo*-like BBB models with the ability to have *in situ* readouts (30, 31). Since microfluidic chips represent physiological fluid flow with realistic dimensions, they can provide technical advantages to improve BBB modeling (32–35). We took advantage of the newly developed advanced organ-on-a-chip devices with microfluidic chips (27, 36–38) to focus on the BBB response to brain-infectious pathogens, in order to get insight on bacterial meningitis in a human-relevant CNS platform (Fig. 1).

Here, we aimed to understand the pathophysiology of two E. coli (ExPEC) strains isolated from NBM. First, we explored the impact of the meningitis E. coli on the morphology and functionality of endothelial, neuronal, and glial cells. Then, we used the neurovascular unit (NVU)-on-a-chip technology to investigate the impact of the pathogens on the individual NVU components (endothelium and astrocytes plus neurons) and on the combined system. Our results showed significant changes in both neuronal and endothelial physiological parameters, due to their interaction with meningitis bacteria.

Our experiments open up a variety of interesting possibilities for studying bacterium-host interactions in a physiologically relevant model. This approach is especially valuable for studies that require large numbers of samples—screening bacterial libraries, plasmids, antimicrobial drugs, or antibodies—which cannot be performed in animal studies. This *in vitro* organ-on-a-chip technology is also effective for genomic and functional genomic studies.

## RESULTS

### Characterization of E. coli strains isolated from NBM.

To shed light on the vascular and neuronal response to pathogenic meningitis bacteria, we used two E. coli strains isolated from patients with newborn meningitis (NBM). These two strains, E. coli O78-285 and O78-287, were serotyped as O78 (39, 40). The choice of these strains offers two advantages over the strains that were used before to study NBM in animal models; they were actually isolated from NBM and they produce a capsule that is different from the K1 capsule of the previously used strains. Strains with K1 capsule have been used because this type of capsule—sialic acid capsule—is the one expressed by *N. meningitis*. The use of bacteria that do not express K1 capsule, but express another capsule, could determine if K1 capsules are the only required capsule for NBM. Sequencing, genetic, and physiological analyses of these strains indicated that they do not produce any toxin or hemolysin (41). Both strains carry a large plasmid, as shown in Fig. S1A in the supplemental material, which was identified as a ColV plasmid (40). This plasmid carries the *aer* gene (41), which codes the production of the siderophore aerobactin and is essential for serum survival (42, 43). Furthermore, we showed that both O78-285 and O78-287 express the *iroB* and *iroC* genes (Fig. S1B), which are also required for overcoming iron deficiency and survival in human blood. Indeed, these two NBM isolates were not inhibited by 40% serum, as shown in Fig. S1C, in contrast to the control E. coli K-12 strain, which was totally inhibited by the serum. The ability to survive serum is critical, as the bacteria must go through the bloodstream in order to get to the meninges and brain.

**FIG 2:**
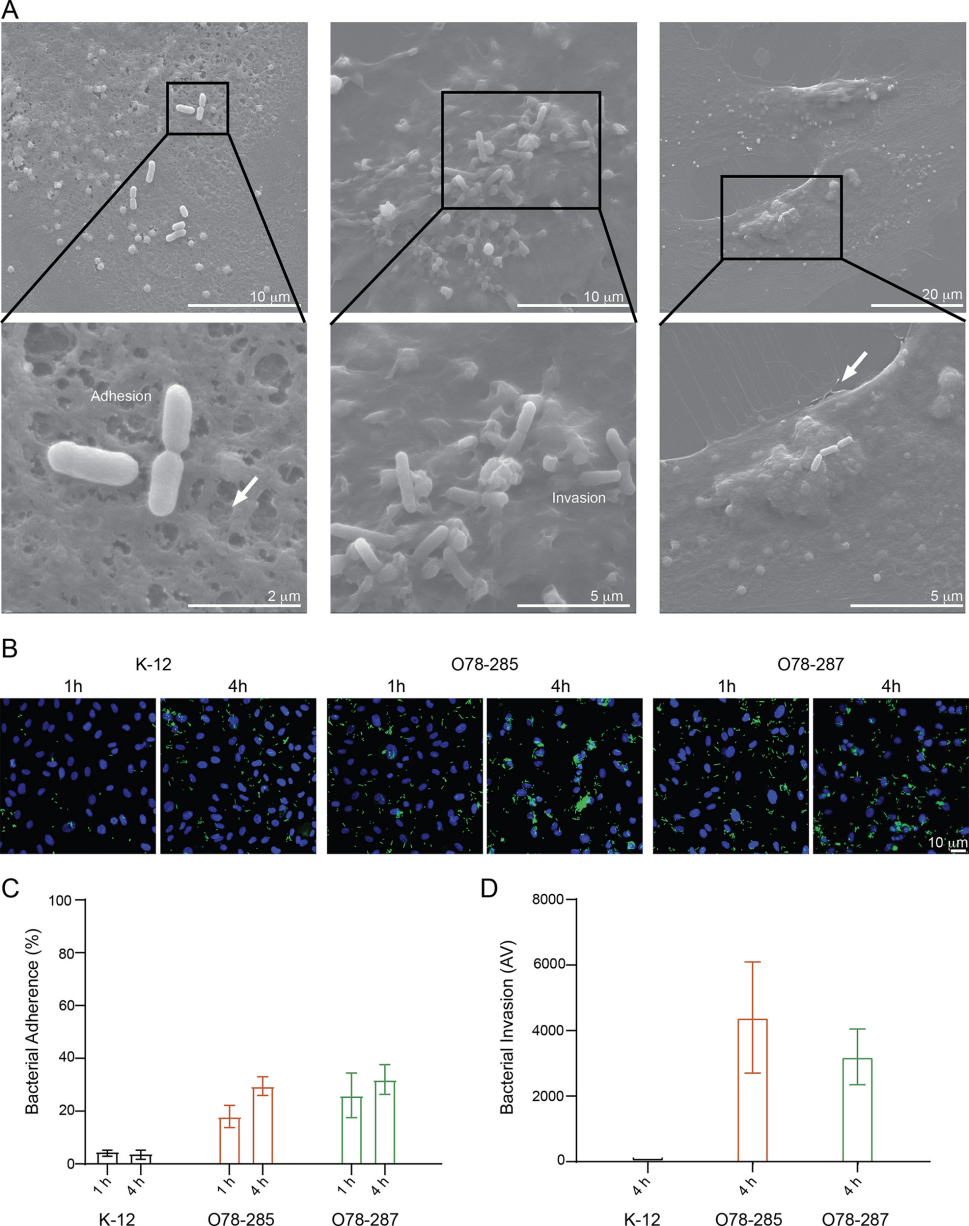
Adherence and invasion of E. coli NBM strains. (A) Representative SEM images at different magnifications showing the adherence and invasion of the pathogenic E. coli bacteria on vascular endothelial cells. (B) Representative confocal images of HUVECs stained for the nuclei (DAPI, in blue) and infected with the bacteria (in green). Scale bar = 10 μm. (C) Analysis of the bacterial adherence. (D) Analysis of the invasion levels determined by the gentamicin protection assay, as described in Materials and Methods.

The ability of the bacteria to adhere to the vascular cells and invade them was assessed in a first set of experiments by scanning electron microscopy (SEM) (Fig. 2A). We showed that the meningitis bacteria adhered to the surface of the vascular cells and penetrated their membrane. Higher-magnification images clearly show that the pathogenic E. coli induce significant cellular damage, evidenced by the formation of pores on the surface of the vascular cells and the disruption of cell-cell junctions (marked by arrows). In order to gain insight into how bacteria adhere to endothelial cells, confocal images were taken at several time points (Fig. 2B) and the percentage of adherence was calculated (Fig. 2C). In human umbilical vein endothelial cells (HUVECs) exposed to the pathogenic bacteria O78-285 and O78-287, we observed a stronger increase in the adherence (O78-285, from 18% ± 4.2% after 1 h to 29.5% ± 3.5% after 4 h; O78-287, from 26% ± 8.5% after 1 h to 32% ± 5.6% after 4 h) compared to the K-12 (from 4.7% ± 0.3% after 1 h to 4% ± 1.4% after 4 h). The bacterial invasion was also determined by the gentamicin-protection assay; for O78-285 and O78-287 the numbers of intracellular bacteria after 4 h were 4.4 · 10^3^ ± 1.6 · 10^3^ (0.073%) and 3.2 · 10^3^ ± 141 (0.057%), respectively (Fig. 2D).

### Effect of the bacteria on endothelial functions.

**FIG 3:**
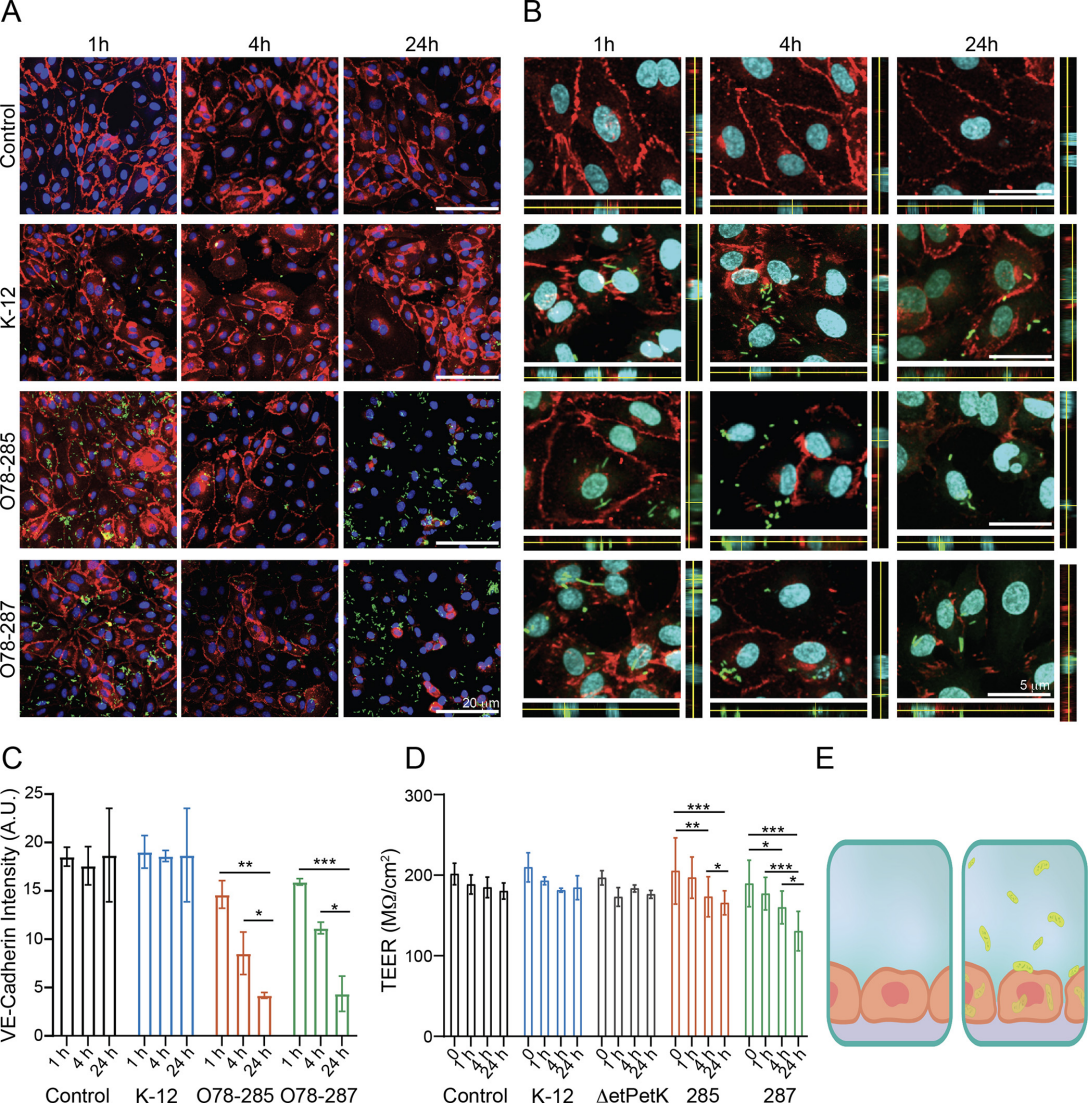
Effect of meningitis E. coli on vascular functionality. Cell cultures were infected with bacteria at an MOI of 10 (10^5^ endothelial cells were infected with 10^6^ bacteria). (A) Confocal reconstructions for VE-cadherin (red) and Hoechst (blue) for the four specified conditions and time points. Scale bar = 20 μm. Note the bacteria in green. (B) High-magnification confocal reconstruction with an orthogonal view for the specified conditions and time points. Scale bar = 5 μm. (C) Analysis of the VE-cadherin expression levels from the images presented in panel A. (D) Changes in barrier functions as a result of meningitis bacteria were assessed by transepithelial/transendothelial electrical resistance (TEER) measurement. Note the statistical differences assessed by F-statistic with a two-way ANOVA test, followed by the Holm-Sidak test for multiple comparisons. (E) Illustration of the endothelium without and with the bacteria.

To better understand the vascular response to the meningitis bacteria, we used several *in vitro* platforms, which included the Transwell platform and insert chips (Fig. 1B). These platforms allowed us to examine essential parameters such as changes in tight junctions and membrane permeability, which are essential functions of the endothelium serving as a barrier. Ideally, one should use brain endothelial cells. However, primary endothelial cells are essentially impossible to include in regular chip design. Therefore, we used human umbilical vein endothelial cells (HUVECs). We and others characterized these cells and found that HUVECs have all the significant tight-junction proteins (Fig. S2A) and can therefore be used to represent endothelial cells (44). HUVECs were cultured on these platforms and infected with the different strains of E. coli, and the effect on endothelial functionality was examined. First, we used immunohistochemistry (IHC) to investigate the expression of VE-cadherin by HUVECs that were incubated with the various E. coli strains at several time points, 1 h, 4 h, and 24 h (Fig. 3A and Fig. S2B). As shown in Fig. 3A to C, the cells exposed to the pathogenic strains, O78-285 and O78-287, showed a significant reduction in VE-cadherin intensity, underlying a potential impact of the bacteria on the properties of the tight-junctions and on the endothelial permeability. The VE-cadherin significantly decreased in HUVECs exposed to O78-285 and O78-287 after 4 h and 24 h (O78-285, from 14.6 ± 1.4 arbitrary units [AU] after 1 h to 8.5 ± 2.2 AU after 4 h and 4.2 ± 0.3 AU after 24 h; O78-287, from 15.9 ± 0.3 AU after 1 h to 4.3 ± 1.8 AU after 24 h; *, *P* < 0.05; **, *P* < 0.01; ***, *P* < 0.001; two-away analysis of variance (ANOVA); two different culture series). It is important to note that no significant effect was observed on either the control or the nonpathogenic (K-12) samples. As the tight junctions are strongly coupled to the barrier function, we used transepithelial/transendothelial resistance (TEER), a standard method that identifies changes in impedance values, which indicated the integrity and permeability of the cell monolayer (44, 45). The TEER measurements confirmed the previous data related to VE-cadherin expression, with a significant decrease in permeability in cells exposed to O78-285 and O78-287 E. coli strains (Fig. 3D). More strongly, the TEER values significantly decreased in HUVECs interfaced with O78-285 and O78-287 after 4 h and 24 h (O78-285, from 205.2 ± 41.2 MΩ/cm^2^ before the infection to 173.2 ± 24.9 MΩ/cm^2^ after 4 h and 165.7 ± 14.8 MΩ/cm^2^ after 24 h; O78-287, from 189.7 ± 28.9 MΩ/cm^2^ before the infection to 160.0 ± 20.5 MΩ/cm^2^ after 4 h and 130.6 ± 24.4 MΩ/cm^2^ after 24 h; *, *P* < 0.05; **, *P* < 0.01; ***, *P* < 0.001; two-away ANOVA; three different culture series). The ability of the pathogenic bacteria to impact barrier permeability was also confirmed by the fluorescein isothiocyanate (FITC)-dextran assay. The fluorescence intensity of FITC-dextran strongly increased in the O78-285 and O78-287 samples (Fig. S2C) compared to the HUVEC control cultures, confirming their capability in impairing the endothelial barrier. Hence, these data show a strong effect of the pathogenic E. coli on endothelial functionality, which could explain the reduction in endothelial integrity.

While it is known that in NBM the bacteria penetrate the BBB and reach the brain parenchyma, it is still unclear by which mechanism this occurs. To address this challenge, we performed high-magnification confocal imaging to determine how meningitis E. coli bacteria enter cells. As shown in Fig. 3B, the two meningitis strains not only destroy tight junctions, but can also enter the cell body. This finding contrasts with the K-12 results, where the tight junctions were intact and most of the bacteria were on the membrane and not inside the cells (Fig. 3E).

### The bacteria increase the inflammatory response.

**FIG 4:**
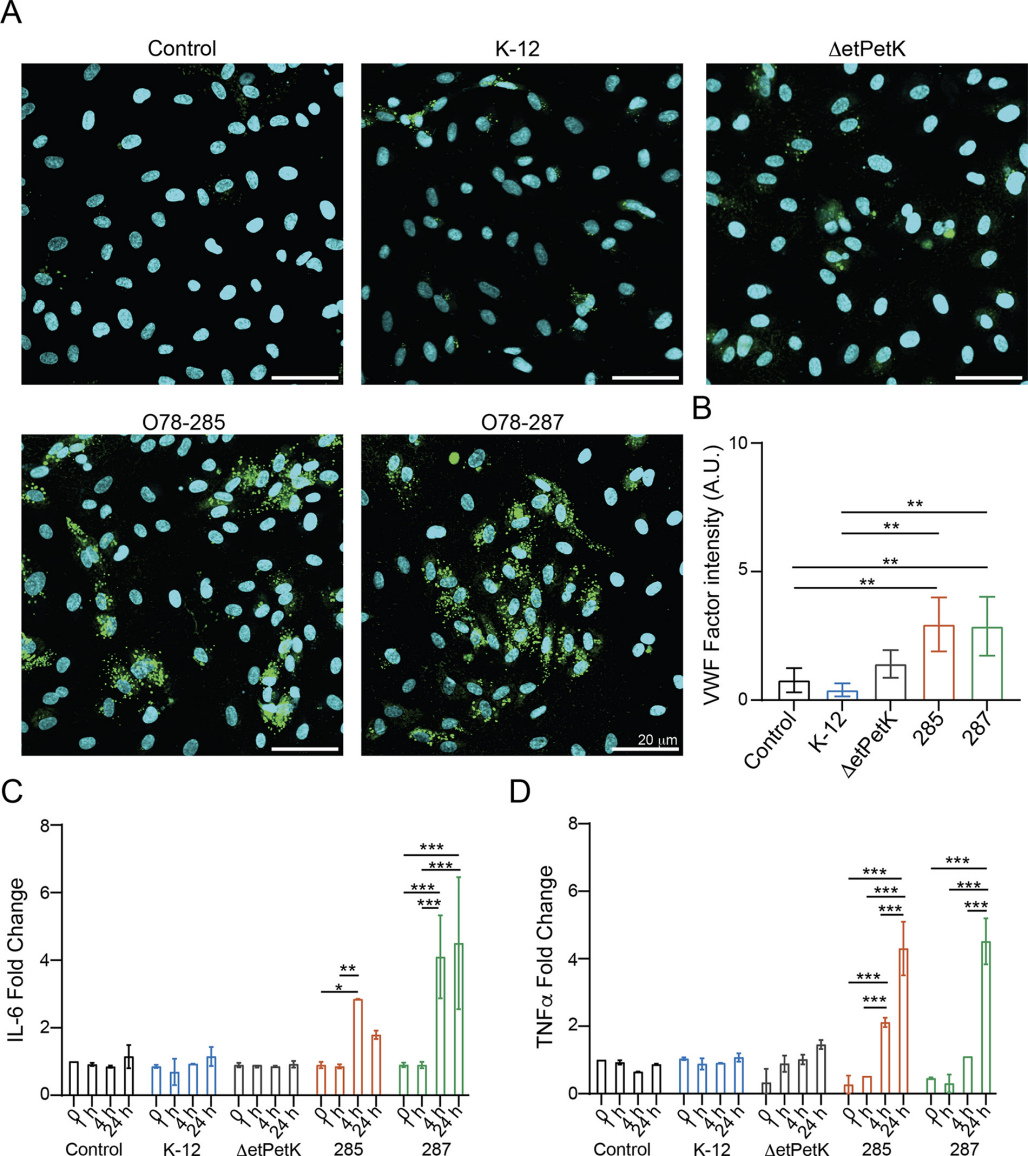
Effect of meningitis E. coli on inflammatory response. The experiments were carried out as described for Fig. 3. (A) Confocal reconstructions of HUVECs stained for Von Willebrand factor (VWF) (green) and Hoechst (blue) under five conditions: control (untreated), K-12, O78-Δ*etPetK*, O78-285, and O78-287. Scale bar = 20 μm. (B) analysis of VWF expression levels. (C) Fold change of interleukin-6 (IL-6). (D) Fold change for TNF-α in response to the different bacteria.

Recently, it was shown that some meningitis E. coli contribute to the increase in inflammatory response and the induction of proinflammatory cytokines (1, 15). To further study the impact of our virulent bacteria in inducing inflammation and increasing coagulation, we stained and analyzed the expression level of Von Willebrand factor (VWF; Fig. 4A and B). Indeed, VWF is an emerging mediator of vascular inflammation, favoring leukocyte recruitment, activating complement cascade, and participating in vascular permeability impairment (46, 47).

Since the strongest effect of the bacteria on vascular permeability was identified after 24 h, we decided to characterize the VWF expression at this specific time point. As shown in Fig. 4B, the control samples (control, K-12) did not exhibit a marked expression of VWF, whereas the cells interfaced with O78-285 and O78-287 showed a significant change. Moreover, as VWF is also associated with increased inflammation (48), we monitored changes in cytokine expression following infection (Fig. 4C). As expected, we observed a significant increase in interleukin-6 (IL-6) and tumor necrosis factor alpha (TNF-α) secretion in the cells exposed to O78-285 and O78-287 (Fig. 4C and D) compared to the control cultures, confirming the ability of the pathogenic strains to induce vascular inflammation.

Bacterial capsules are a critical virulence factor in many infections (49). It was previously shown that the K1 capsule is essential for E. coli meningitis (50, 51). E. coli serotype O78 has a group 4 (O-antigen) capsule (50) which was shown to be essential for serum resistance (52). The fact that our meningitis strains have a non-K1 capsule enabled us to determine if the requirement for capsule is general or only specific to sialic acid (K1 capsules). Indeed, we showed that the O-antigen, group 4, capsule is also essential for inducing the inflammatory response. As shown in Fig. 4, bacteria with the *etKetP* genes, which code the synthesis of group 4 capsule, deleted (Δ*etPetK*) behave like the control and the K-12 bacteria in respect to the inflammatory response (Fig. 4).

### Interaction of bacteria with neuronal cultures.

**FIG 5:**
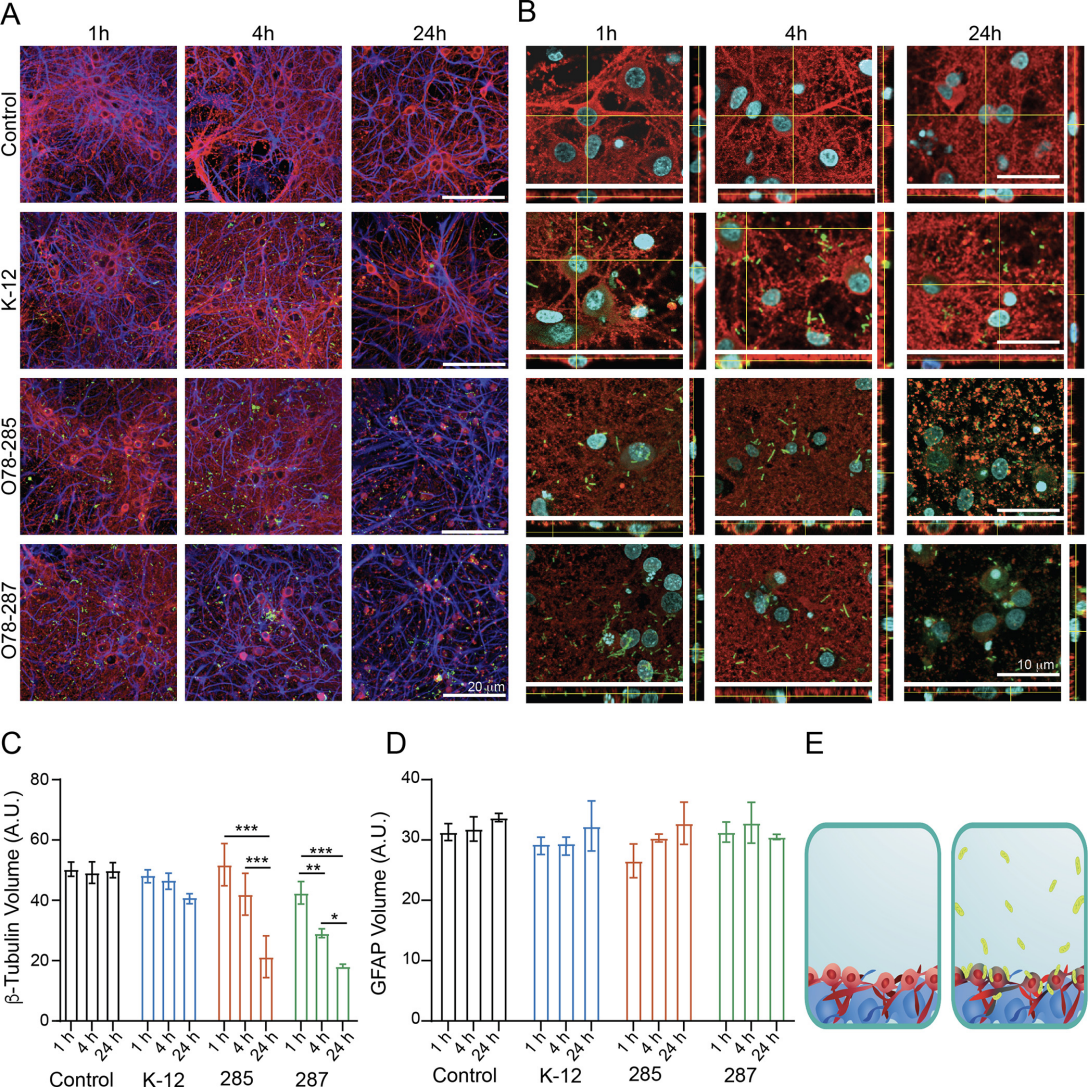
Effect of meningitis E. coli on neuroglia morphology. The experiment was carried out as described for Fig. 3. A total of 10^4^ cells were infected with 10^5^ bacteria. (A) confocal reconstructions for β-tubulin (red) and GFAP (cyan) for the four specified conditions and time points. Scale bar = 20 μm. Note the bacteria in green. (B) High-magnification confocal reconstruction with orthogonal view for the specified conditions and time points. Scale bar = 10 μm. (C) Changes in β-tubulin volume as a result of meningitis bacteria. (D) Changes in GFAP volume as a result of meningitis bacteria. (E) Illustration of the neurons and astrocytes without and with the bacteria.

Once we characterized the impact of the meningitis E. coli strains on the morphology and functionality of the vascular cells, we continued to the next physiological system that is affected by NBM, which is the brain parenchyma. To do so, we used cortical neurons isolated and cultured for 14 to 20 days *in vitro* (DIV). Primary neuronal cultures were incubated for 1 h, 4 h, and 24 h with the different E. coli strains, and their morphology and functionality were investigated via confocal microscopy and electrophysiological measurements. First, we determined the cellular composition of control and E. coli-treated cortical cultures using immunofluorescence markers for astrocytes (GFAP) and neurons (β-tubulin III). We identified both β-tubulin III (neuronal cells) and GFAP (a stain for glial fibrillary acidic protein) under all conditions at the three different time points (Fig. 5A). Once the pathogenic bacteria were added, the β-tubulin III volume was significantly reduced after 24 h in the O78-285 and O78-287 samples and even after 4 h for the O78-287 strain (Fig. 5A to C). No differences were detected for the area of the glial cells (Fig. 5D). Thus, the pathogenic strains specifically altered neuronal morphology and survival. It is important to note that, in contrast to the endothelial cells, the meningitis bacteria did not enter the neurons or the astrocytes, as was shown in the high-magnification imaging of the neuronal-glial culture (Fig. 5B), and were mainly bound to the neuronal membrane (Fig. 5E).

**FIG 6:**
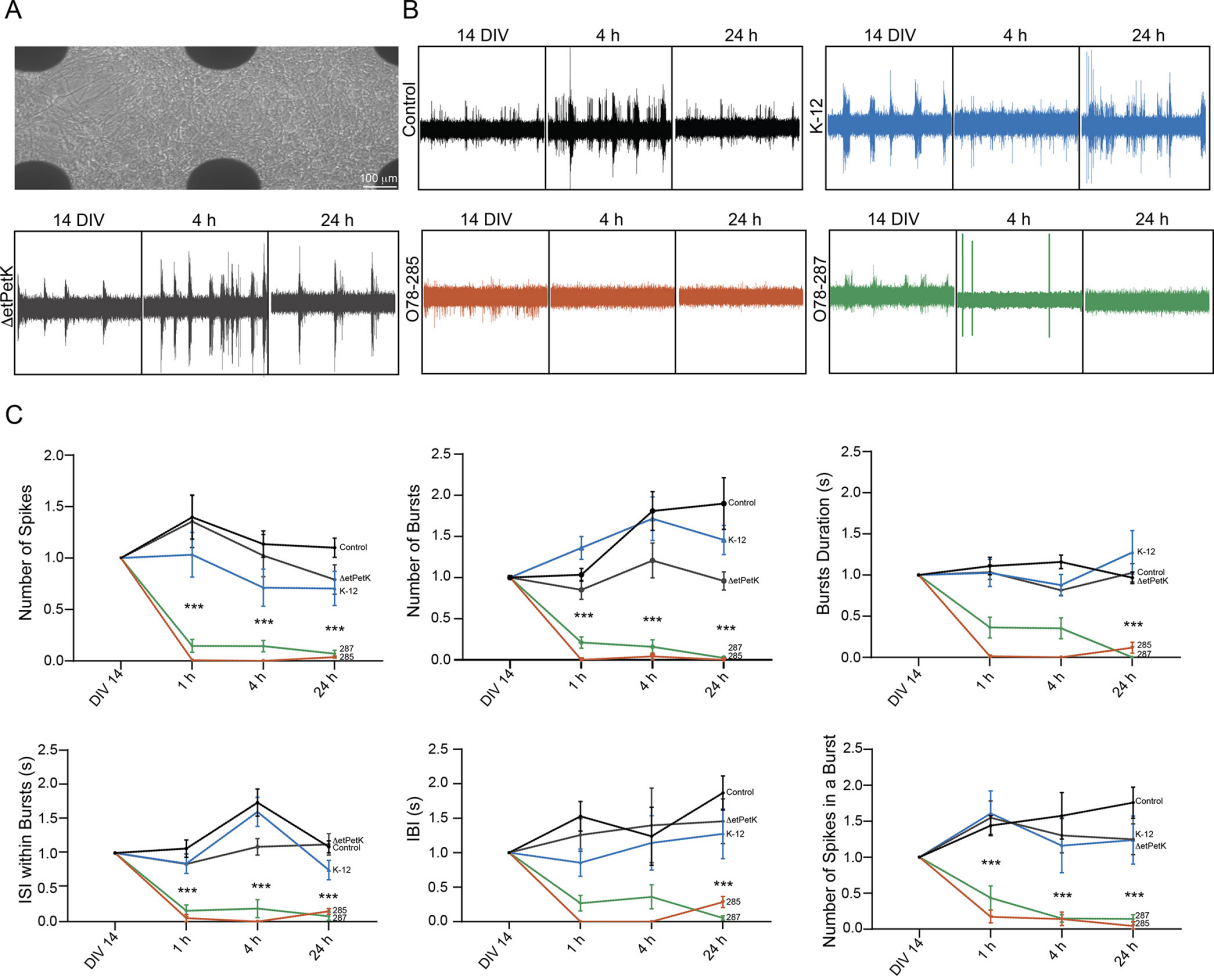
Effect of meningitis E. coli on neuronal functionality. (A) Bright-field image of cortical neurons grown on the MEA platform. Scale bar = 100 μm. (B) Representative electrophysiological recordings of the different conditions at three different time points (14 DIV, 4 h, and 24 h). (C) Plots showing different electrophysiological parameters for the different conditions at four different time points of neurons directly infected with the pathogenic bacteria (*P* values are shown in Table S1).

Once the neuronal morphology and survival were characterized, we sought to identify how the neuronal functionality changes due to the bacterial infection (Fig. 6). For this purpose, we employed a multielectrode array (MEA) recording system (Fig. 6A) to record extracellular spontaneous spikes in primary cortical neuronal cultures. Since this system allows recording and analysis from the same cultures before and after the treatment, the variability between cultures is greatly diminished. Figure 6B shows representative current tracings of the recorded electrical activity for the different strains at 3 time points. The appearance of spikes provided clear evidence of functional synapse formation, which is a widely accepted index of network efficacy. Moreover, it can clearly be seen that the O78-285 and O78-287 pathogenic strains significantly diminished neuronal electrical activity (Fig. S3). Quantification of more than 9 electrodes out of 12 per sample showed that these strains significantly decrease the neuronal activity in several parameters, such as the pattern of spontaneous spikes and the burst activity and properties (Fig. 6C). We determined the burst activity as a series of spontaneous spikes which occurred within a short period of time. We defined a minimum of 3 spontaneous spikes with a maximum interspike interval of 0.3 s as a burst, and showed that the pathogenic bacteria can significantly impact burst activity (Fig. 6C). The strong decrease in synaptic electrical activity strongly confirmed the significant reduction in β-tubulin III described previously, emphasizing the ability of E. coli O78-285 and O78-287 to significantly alter synaptic formation and function, affecting cell survival and the proper functionality network size.

### Construction of a neurovascular-unit-on-a-chip.

**FIG 7:**
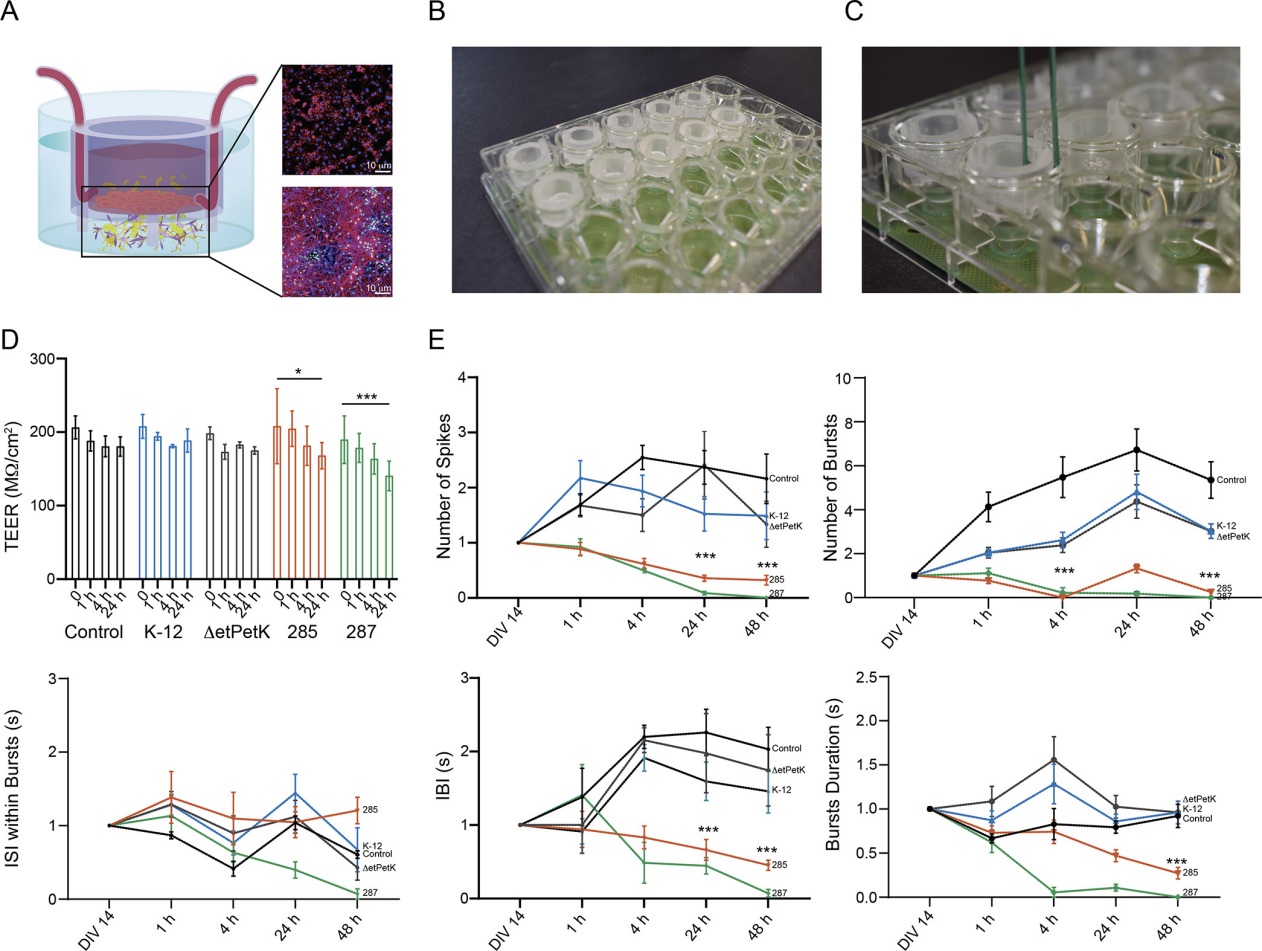
Effect of meningitis E. coli on vascular-neuronal coculture functionality. (A) Sketch representing the insert chip used in this work with a coculture of vascular cells on the top of the membrane and neurons on the top of the MEA. The confocal reconstructions show the two kinds of cells: the vascular (stained for VE-Cadherin [red] and Hoechst [blue]) and neuroglia cells (stained for β-tubulin [red], GFAP [cyan], and Hoechst [blue]). Scale bar = 10 μm. (B) Picture showing the insert chip inserted in the MEA platform. (C) Picture showing the insert chip in the MEA with the ability to perform TEER and electrophysiological measurements simultaneously. (D) Changes in barrier functions of HUVECs cocultured with the neurons and infected with the different bacteria. (E) Plots showing different electrophysiological parameters for the different conditions at four different time points, with only the HUVECs infected with the bacteria (*P* values are shown in Table S1).

In order to have deeper understanding of the effect of the meningitis bacteria on the brain, we constructed an organ-on-a-chip to emulate the neurovascular unit (NVU). This system mimics the response of the whole system, which could be different from the response of the independent components (endothelial and neuronal separately). To do so, we cocultured vasculature and neuronal cells on an insert chip (30). This insert chip is 3D-printed from clear dental resin, with a single porous membrane on which cells can be cultured and positioned near its base (Fig. 7A). Importantly, the insert chip was integrated into a commercial MEA platform (Fig. 7B), such that the permeability of the barrier tissue was measured using the commercial TEER system, while the electrical activity was measured via the MEA platform (Fig. 7C). The NVU was mimicked as follows. HUVECs were cultured on the insert chip; when the cells created a confluent monolayer, the chip was placed on top of the MEA and cultured with cortical neurons for 14 DIV, and the bacteria were added on the vascular compartment, similar to the *in vivo* scenario (Fig. 7A). In this way, we were able to better recapitulate the NVU structure and investigate the bacterial impact on neurons after the E. coli was added on the endothelial side. Barrier permeability was measured with TEER (Fig. 7C and D), together with neuronal electrical activity (Fig. 7C and E). As shown before for the vascular cells, the meningitis bacteria significantly impaired endothelial permeability after 4 h (Fig. 7D). However, in contrast to the previous experiments in the independent neuronal culture, where a significant decrease in the neuronal activity was observed mainly after 4 h, here, we see that the endothelial layer postponed the decrease in the electrical activity in about 20 h (Fig. 7E and Fig. S4). These results suggest that when the meningitis bacteria were added to the vascular side, they penetrated the endothelium and then reached the neurons, where they significantly decreased the spike and burst activity. It is important to note that the dynamic by which the neuronal electrical activity is affected by the bacteria in the coculture is very different from the decay in the independent neuronal culture. In the coculture, the decrease is much more gradual, and it can take up to 48 h to see an effect in some of the parameters (e.g., burst duration), in comparison to the independent neuronal culture, where the decay is much faster.

## DISCUSSION

Here, we described a study of E. coli NBM using *in vitro* models of endothelial cells, neurons, and a neurovascular-unit-on-a-chip (NVU-on-a-chip), which is composed of cocultured vasculature and neuronal cells on an insert chip (30). With these systems, it is possible to determine the effect of the bacteria on basic cellular physiological parameters, such as membrane stability, the fate of the tight junctions, and the localization of the bacteria on or in the cells. These parameters cannot be determined in animal studies, which use infant rodents or pigs as models for NBM. Moreover, the use of NVU-on-a-chip enables the study of the infection in a model system that includes several brain tissues and the interactions between them. For example, while in independent neuronal cultures a significant decrease in neuronal activity was observed after 4 h, the presence of the endothelial layer delayed the decrease in neuronal activity by many hours. The presence of the endothelial layer also affects the dynamic of the neural electrical activity, which decreases much more gradually up to 48 h—than in independent neuronal cultures.

Having a good model system is essential for understanding bacterial meningitis, especially if we consider that inadequate knowledge of the pathogenesis of E. coli meningitis, as well as how the bacteria penetrate the BBB, has contributed to the significant mortality associated with this disease.

The other essential parameter is the understanding of the bacteria—which genetic and physiological features are essential for causing meningitis? For our experiments, we used two strains of E. coli O78, O78-287 and O78-285, which were isolated from NBM (39, 40). They carry the ColV plasmid and several genes coding iron uptake (Fig. S1) which are essential for surviving in the bloodstream and enable bacteria to reach the meningi and the brain. We assumed that genes involved in the biosynthesis of capsules should be important, as capsules are important in the pathogenesis, and moreover, several studies indicated the importance of the K-1 capsule for NBM. Indeed, a mutant we constructed that is deleted for the *etPetK* genes, coding for capsule formation in E. coli O78, did not cause any damage in our *in vitro* systems. The finding that in our *in vitro* system we can detect mutants that do not cause infection opens up the possibility of carrying out genomic experiments aimed at identifying the genes which are essential for NBM. Thus, in our system it is possible to screen large libraries of mutants, in contrast to work with newborn mice, where the number of samples is limited. Moreover, the system makes it possible to perform functional genomic experiments, screening for transcription and translation patterns of the bacteria and the tissue culture cells.

The most critical step in the pathogenesis of bacterial meningitis is the penetration of the extracellular pathogens across the BBB, a formidable defense system that normally keeps out pathogens and toxins. The best model system for this study would be brain endothelial cells. However, this could not be done in our chip design. Instead, we used human umbilical vein endothelial cells (HUVECs), which are an acceptable substitute, as they have all the significant tight-junction proteins and can represent endothelial cells (Fig. S2) (44). Indeed, the HUVEC model is described as physiologically representative of the BBB in different studies (35, 53, 54), allowing the study of the physiological and pathological conditions as well as the effects of novel drugs on the BBB. Our study confirms previous results demonstrating that the virulent E. coli invade the vascular cells, a property that is a prerequisite for penetration into the brain (22, 55). Furthermore, the confocal measurements show the ability of the pathogenic strains to cross the BBB both through the transcellular method, where they traverse through the endothelial cells themselves, and through the paracellular method, disrupting the tight junctions. Once they have crossed the barrier, the pathogenic strains exit through the other side of the vascular cell that is in direct contact *in vivo* with astrocytes and neurons.

As previously evidenced in E. coli-induced meningitis, cytokines and chemokines potentially contributed to BBB damage (1, 56). We confirmed these results, emphasizing the potential ability of the bursts of proinflammatory cytokines, such as TNF-α and IL-6, in leading directly to dysfunction of the endothelial barrier and an increase in vascular permeability in the brain, thus finally leading to severe CNS injury (57, 58). An open question is how the meningitis bacteria enter the brain and impact the neurons (59). Although the specific molecular mechanism behind the interaction between such pathogens with neurons is still under investigation, it is clear that bacterial interaction with neurons and neuroinflammatory responses within the brain lead to neuronal cell death. Recent studies suggest that the bacteria damage the tight junctions, which allows the bacteria to penetrate the brain parenchyma. Here, we support these findings, as we see that both VE-Cadherin and the TEER values significantly decrease. However, our results suggest that in addition to this mechanism, the bacteria can enter the endothelium and go through the cells. It is important to note that the tight-junction disruption and the invasion of the neural cells occur only with the meningitis strains and not with the avirulent K-12 strain. Thus, commensal bacteria, such as K-12, did not produce any damage, and the cells treated with them behaved like the untreated control cells. This finding stresses the need for genome-wide identification of the genes which are responsible for NBM, as stated before.

The interaction of the meningitis bacteria with the neuronal cultures revealed a number of interesting points. (i) The neurons are much more susceptible to the meningitis bacteria than the glial cells (astrocytes). (ii) The effects occur only with the meningitis bacteria and not with the avirulent K-12 strain. (iii) There is a significant difference in the interaction of the bacteria with the endothelium and the neurons. While the bacteria can penetrate the endothelium, we could not observe such processes inside the neurons.

While it is known that NBM can have major effects on the neonate brain, which can lead to major disabilities and even death (59), it is not known how the meningitis bacteria interact with and affect the neurons. One hypothesis is that bacterial meningitis can cause neuroinflammation (2), promoting neuronal damage, which might have an unrepairable effect on neuronal circuits due to the postmitotic state of neurons (59, 60). Indeed, neuroinflammation causes the release of several cytotoxic compounds, including reactive oxidative species and nitric oxide, which can stimulate the release of proapoptotic compounds, ultimately leading to apoptosis of neurons and other brain-resident cells (61). Because neurons are in a postmitotic state, this has potentially deleterious effects, as it contributes to neuronal degradation without future replacement of cells (60). Our study demonstrates that within a very short time (less than 24 h) the neurons are significantly damaged and lose their activity.

Once we integrated the endothelium with the neurons to create an NVU-on-a-chip system, we could show that the interaction of the bacteria with the combined system was similar to what was observed with the individual compartments (only endothelium or only neurons). However, the dynamic of the process was different; the effect on the neurons was delayed by about 20 h. This finding demonstrates that the endothelial layer protects the neuron by stalling the bacteria, but it does not stop them from entering the brain parenchyma and eliminating the neuronal functionality.

Another interesting observation was that the dynamic of the change in the neuronal electrical activity is different between the independent cultures and the cocultures. The main reason for this is the gradual entrance of the meningitis bacteria through the endothelium, as opposed to the independent neuronal cultures, where all the meningitis bacteria are added at once. This result can confirm the previous assumptions that the bacteria enter the BBB, increase the release of cytotoxic compounds and in this way lead to neuronal damage (59).

In conclusion, the availability of a model system that mimics the *in vivo* neuron-endothelial cell interactions enables the study of NBM in terms of the physiological effects on the brain tissues and enables a genome-wide study of the infecting bacteria. Such studies should further our understanding of this infection and the bacteria involved. Moreover, the system can be adapted to study the pathophysiology of additional infections involving the central nervous system.

## MATERIALS AND METHODS

### Strains, growth conditions, and media.

All the E. coli strains used in this study are listed in Table 1. The bacteria were grown with aeration at 37°C in Luria-Bertani (LB) broth (Difco). Chloramphenicol (Sigma-Aldrich) was added, when required, at a final concentration of 33 μg/mL. Transformation of pTac-YFP (see Table 1) was carried out by electroporation using the standard protocol (62, 63). Overnight cultures were diluted 1:100 in LB medium, and turbidity at an optical density at 600 nm (OD_600_) was measured every 20 min in a BioTek Eon plate reader. Sterile and filtered human male AB plasma (Sigma-Aldrich) was used when studying the effect of serum. For infection, fresh overnight cultures were diluted and grown to the mid-log phase (OD_600 nm_ = 0.6). They were then diluted to give a multiplicity of infection (MOI) of 10. This MOI was chosen in order to ensure that all the cells were infected but to not overload the cultures with bacteria. Invading bacteria were quantified by a standard antibiotic protection assay, as previously described (64, 65).

**TABLE 1:** Strains and plasmid used in this study

Plasmid or strain	Description	Reference
K-12	*recA*, *endA*, *lacZΔM15*	cgsc2.biology.yale.edu
O78-285	Isolated from NBM	39
O78-287	Isolated from NBM	39
O78-Δ*etPetK*	Deletion mutant of the *etPetK* genes	52
pTac-YFP	Cap^r^, Kana^r^, yellow fluorescent protein	73

### DNA techniques.

Isolation of DNA and agarose gel electrophoresis were performed as previously described (66). Large plasmids were prepared and visualized as previously described (67). The *iroB* and *iroC* genes were detected by PCR using the primers described in Table S2.

### Endothelial cell cultures.

Human umbilical vein endothelial cells (HUVECs) (PromoCell GmbH, Heidelberg, Germany) were used to test the effect of the bacteria on vascular properties. After thawing, the frozen HUVECs were expanded in low-serum endothelial cell growth medium (PromoCell) at 37°C with 5% CO_2_ in a humidifying incubator and used at passages p3 to p6 (30, 44). Cells were grown to 80 to 90% confluence before being transferred to transparent polyethylene terephthalate (PET) Transwell supports (0.4 μm pore size, Greiner Bio-One, Austria), to a plastic well plate (Corning), to a glass-bottom well plate (Cellvis, Mountain View, CA), and to the insert chip (30). Before seeding, the uncoated substrates were treated with entactin-collagen IV-laminin (ECL) cell attachment matrix (Merck) diluted in Dulbecco’s modified Eagle’s medium (DMEM) (10 μg/cm^2^) for 1 h in the incubator. Then, the HUVECs, harvested with trypsin/EDTA solution (Biological Industries), were seeded inside the culture platforms at a density of 250,000 cells/cm^2^ and grown for 48 h. Then, bacteria were added and their effect on cell behavior was tested after 1 h, 4 h, and 24 h.

### Neuronal cell cultures.

Primary dissociated cultures were obtained from postnatal rats (p2 to p3) as previously described (68, 69). All experiments were approved by the local authority and performed in accordance with Israeli law. All efforts were made to minimize animal suffering and to reduce the number of animals used. Neuronal hippocampal cells were plated on a glass-bottom-well plate (Cellvis) and on a multielectrode array (MEAs; Multi-Channel Systems, Reutlingen, Germany) platform for network investigation. Prior to cell seeding, the glass-bottom plates were treated with poly-d-lysine, while the MEA substrates were treated with polyethyleneimine (PEI, Sigma-Aldrich) in Borate buffer (Sigma-Aldrich) overnight at 4°C. Then, both substrates were rinsed 4 times with distilled water, sterilized with UV light for 1 h, and treated with laminin (20 μg/mL, Sigma-Aldrich) diluted in a plating medium containing neurobasal medium (Gibco), supplemented with fetal bovine serum (FBS, 5%, Biological Industries), B27 (2%, Gibco), Glutamax (1%, Gibco) and PSA (1%, Biological Industries), for 4 h at 37°C.

Neuronal hippocampal cells were then plated on the coated substrates in a plating medium and incubated at 37°C in a humidified atmosphere enriched with 5% CO_2_. After 24 h had passed since seeding, the medium was replaced (80%) with serum-free neurobasal medium, supplemented with B27 (2%), Glutamax (1%), PSA (1%), and gentamycin (1%, Gibco) (70, 71). Culture medium was renewed (50%) every 3 days from seeding. Plating was carried out at a nominal density of 100,000 cells/cm^2^. Cultures were then used for experiments after 12 to 20 days *in vitro* (DIV).

### Scanning electron microscopy.

Bacterial morphologies and interaction with endothelial cells were qualitatively assessed through scanning electron microscopy (SEM). Images were acquired by collecting secondary electrons on a Quanta 200FEG environmental SEM (ESEM) at high-vacuum mode. The different samples were imaged at 12.5 kV e-beam. Cellular samples were fixed with 2.5% glutaraldehyde (Sigma-Aldrich) and 4% paraformaldehyde (Sigma-Aldrich) in sodium cacodylate buffer for 60 min at room temperature. After 3 washes in cacodylate buffer, samples were postfixed with OsO_4_ in distilled water for 60 min, followed by a dehydration process of dipping the sample in water/ethanol solutions at progressively higher alcohol concentrations (25%, 50%, 75%, 95%, and 100% ethanol for 5 min each). Samples were dried at room temperature. Prior to SEM imaging, samples were sputtered using Au/Pd sputter coater.

### Confocal live imaging.

HUVECs were cultured on glass-bottom-well plates (Cellvis) and incubated with DAPI (4′,6-diamidino-2-phenylindole) in phosphate-buffered saline (PBS) for 10 min at room temperature (RT) to stain the nuclei. Then bacteria were added and confocal images were taken at different time points (1h, 4 h, and 24 h) using an inverted confocal microscope (Olympus FV3000-IX83) with suitable filter cubes and equipped with a 20× (0.8 numerical aperture [NA]) lens objective. Image reconstruction and analysis were done using open-source ImageJ software (72).

### Immunofluorescence and confocal imaging.

Both HUVECs and neuronal cells were rinsed in phosphate-buffered saline (PBS) and fixed in 4% paraformaldehyde (Sigma-Aldrich, Rehovot, Israel) for 20 min at room temperature (RT) 1 h, 4 h, and 24 h after bacterial addition. Immunocytochemistry was carried out after permeabilization with 0.1% Triton X-100 (Sigma-Aldrich) in PBS for 10 min at RT and blocking for 30 min with 5% FBS and 5% bovine serum albumin (BSA) in PBS. The following primary antibodies were applied overnight in PBS at 4°C: rabbit anti-VWF (Abcam, Cambridge, UK), rabbit anti-VE-cadherin (Cell Signaling Technology, Danvers, MA), rabbit anti-CD31 (Abcam) against platelet endothelial cell adhesion molecule 1 (PECAM1), rabbit anti-β-catenin (Cell Signaling Technology), rabbit anti ZO-1 (Cell Signaling Technology), rabbit anti-occludin (Cell Signaling Technology), rabbit anti β-tubulin III (Sigma-Aldrich), and mouse anti-GFAP (Sigma-Aldrich). Cells were then washed three times in PBS and stained with the secondary antibody, anti-rabbit Alexa Fluor 488 (Invitrogen, Carlsbad, CA), anti-rabbit Alexa Fluor 594 (Invitrogen), and anti-mouse Alexa Fluor 647 (Invitrogen) for 1 h at RT. After four washes with PBS, cells were incubated with Hoechst in PBS for 10 min at RT to stain the nuclei. After two washes with PBS, imaging was carried out using an inverted confocal microscope (Olympus FV3000-IX83) with suitable filter cubes and equipped with 20× (0.8 NA), 40× (0.95 NA), and 60× (1.42 NA) lens objectives. Image reconstruction and analysis were done using open-source ImageJ software (72).

### Transepithelial/transendothelial electrical resistance (TEER) measurements.

The barrier properties of the endothelial monolayer were evaluated using TEER measurements 1 h, 4 h, and 24 h after bacterial infection. TEER was measured with the Millicell ERS-2 voltohmmeter (Merck Millipore). TEER values (Ω cm^2^) were calculated at different time points among cells exposed to the different bacterial strains, subtracting the values obtained from a Transwell insert or insert chip without cells, considered as a blank, in three different individual experiments.

### Permeability assay.

HUVECs were cultured on 12-well Transwell inserts as described above. After 4 h and 24 h from the bacterial infection, fluorescein isothiocyanate (FITC)-dextran (Sigma-Aldrich) was administered to the upper compartment of the insert. Then, 1 h after the dextran was added, the fluorescence intensity of the medium in the lower compartment was measured with a fluorescence plate reader at an excitation of 492 nm and emission of 518 nm. The FITC-conjugated dextran concentration obtained from the different conditions was normalized to that obtained from a Transwells (TWs) without cells, considered blank.

### MEA recording.

Neuronal network extracellular recordings were carried out using the 24-multiwell MEA plate system (Multi Channel Systems). Primary cortical cultures were plated on gold-coated electrode MEAs with 12 electrodes (30 μm electrode diameter, 300 μm interelectrode spacing). Raw data were monitored and recorded using the commercial software Multiwell-Screen (Multi Channel Systems), at 37°C in the presence of cell culture medium. The recorded events were analyzed offline with Matlab software.

### Cytokine assay.

A quantitative enzyme-linked immunosorbent assay (ELISA) for IL-6 and TNF-α was performed on conditioned medium of infected HUVECs before the infection and 1 h, 4 h, and 24 h post-bacterial infection, according to the manufacturer’s recommendations (PeproTech Rehovot, Israel).

### Statistical analysis.

The results are presented as the mean ± standard deviation (SD). Statistically significant differences among multiple groups were evaluated by two-way ANOVA, followed by the Holm-Sidak test for multiple comparisons (GraphPad Prism 8.4.3). A statistically significant difference between two data sets was assessed and a *P* value of <0.05 was considered statistically significant.

### Ethics.

All experiments were approved by the local veterinary authority and the animal ethics committee of Tel Aviv University (ethics approval number 01-19-079) and performed in accordance with Israeli law. All efforts were made to minimize animal suffering and to reduce the number of animals used.

### Data availability.

The data that support the findings of this study are available in File S1.
