# Supplementary material for: Insight on Bacterial Newborn Meningitis Using a Neurovascular-Unit-on-a-Chip
Source: Microbiol Spectr. 2023 May 24;11(3):e01233-23. doi: 10.1128/spectrum.01233-23 (PMC10269748; doi:10.1128/spectrum.01233-23)
Supplement: Supplemental file 1 — Supplemental material. Download spectrum.01233-23-s0001.pdf, PDF file, 2.0 MB [file spectrum.01233-23-s0001.pdf]

Supplementary Materials for

Insight on Bacterial Newborn Meningitis Using a Neurovascular-Unit-on-a-Chip

Rossana Rauti[1,2, †], Sharon Novak[3, †], Dvora Biran[3], Keshet Tadmor[4], Yael Leichtmann-Bardoogo[1], Eliora Z. Ron[3], Ben M. Maoz[*1,4,5]

[†] These authors equally contributed
[*]Correspondence: bmaoz@tauex.tau.ac.il

This PDF file includes:

Figs. S1 to S4
Table S1, S2

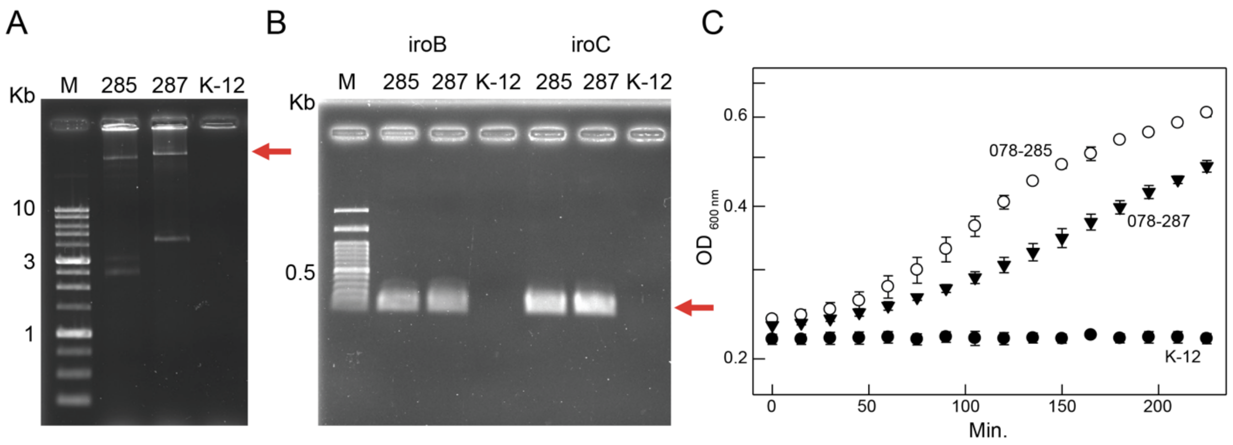

Fig. S1. A) Presence of the ColV plasmid. DNA was obtained from *E. coli* O78-287, O78-285 and K-12 and run on gel electrophoresis, as described in Materials and Methods; **B)** The presence of the *iroB* and *iroC* genes shown by PCR. The DNA was extracted as described and PCR was performed using the specific primers for the *iro* genes (**Table S2**). The PCR products were visualized after gel electrophoresis; **C)** Growth of strains with serum. Overnight cultures were diluted 1:100 and incubated at 37°C with aeration until they reached OD 600nm =0.4. They were then divided into wells in a 96 microtiter plate and serum was added to a final concentration of 40%. Growth was monitored as described in Materials and Methods.

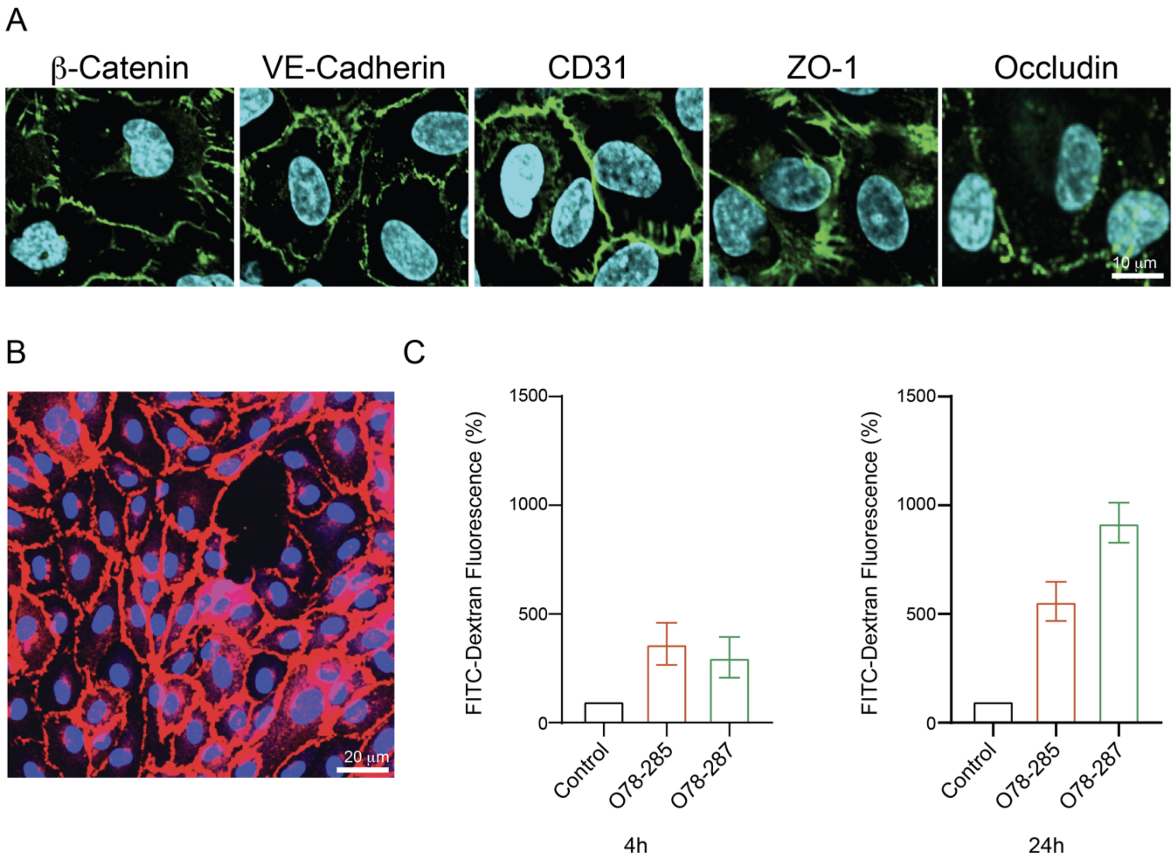

Fig. S2. A) Confocal reconstructions showing the β-Catenin, VE-Cadherin, CD31, ZO-1 and Occludin (green) tight and adherens-junctions' proteins expressions in HUVEC cells. Nuclei are visualized in blue. **B)** Confocal reconstruction showing VE-Cadherin expression (in red) in control condition. **C)** Analysis of the FITC-Dextran fluorescence intensity in HUVEC cells exposed to the pathogenic bacteria compared to the control condition.

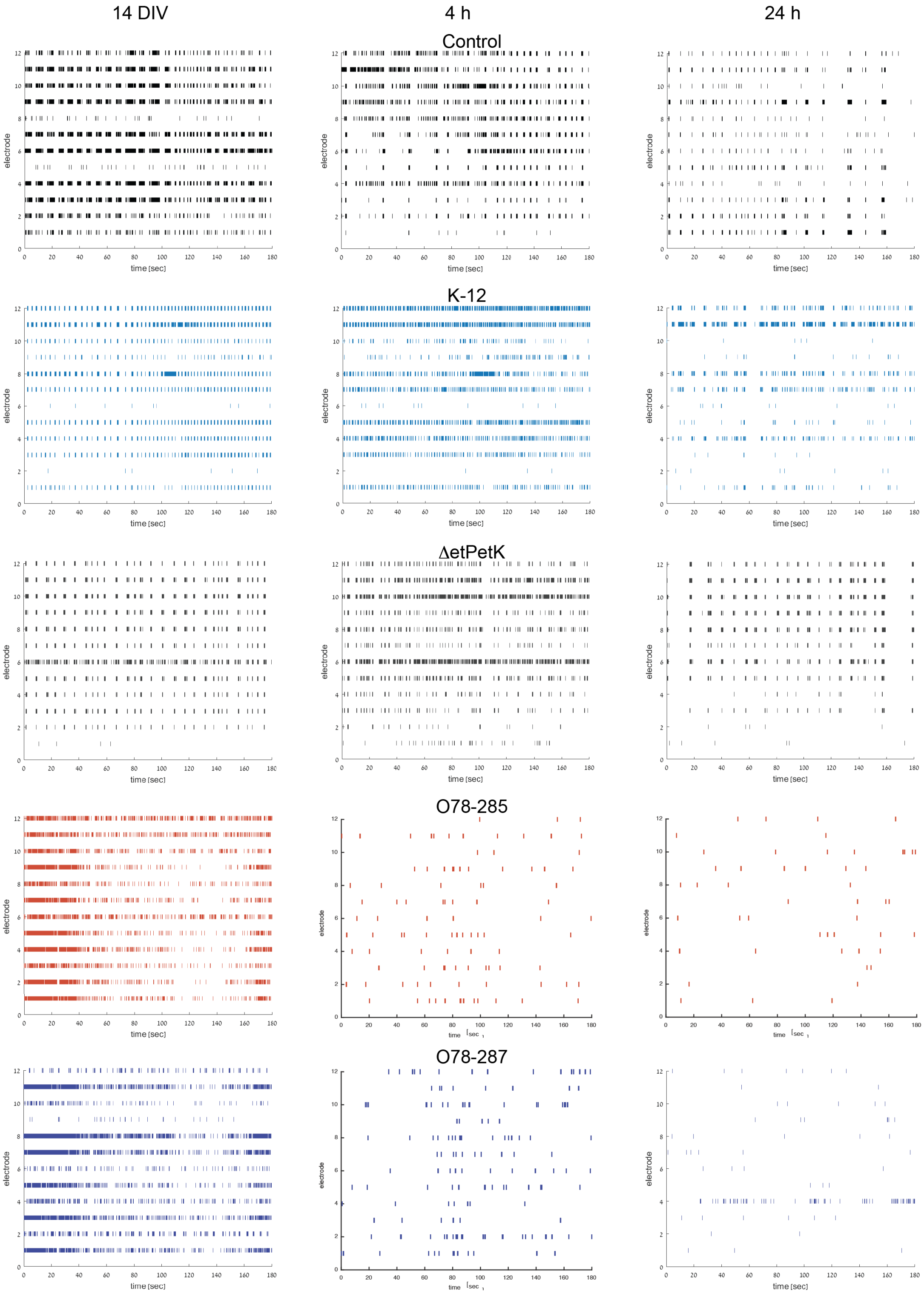

Fig. S3 Raster plots of one representative MEA recordings of the different conditions at three different time points (14 DIV, 4h and 24h post bacterial infection). Each line represents the signal detected by a single electrode of the MEA array, during 180 s recordings.

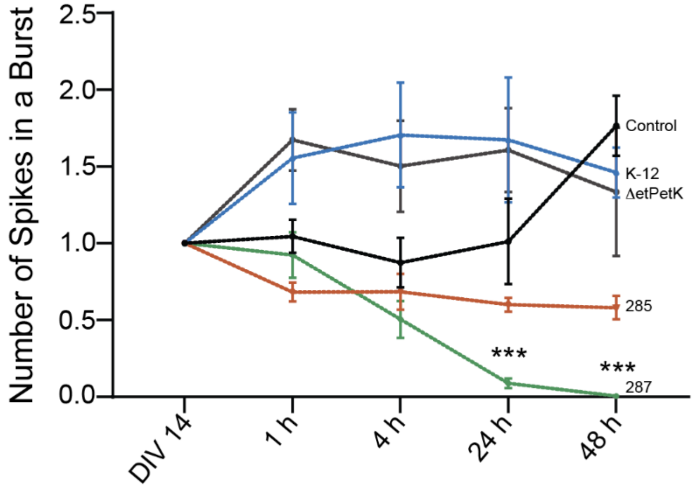

Fig. S4. Plot showing the electrophysiological parameter for the different conditions at four different time points, with only the HUVEC infected with the bacteria (**Table S1**).

Number of spikes	1h	4h	24h
285 coupled vs 285 uncoupled	P<0.05	P<0.05	
287 coupled vs 287 uncoupled	P<0.05		

Number of Bursts	1h	4h	24h
285 coupled vs 285 uncoupled			P<0.05

Bursts Duration	1h	4h	24h
285 coupled vs 285 uncoupled		P<0.05	P<0.05
287 coupled vs 287 uncoupled		P<0.05	

Table S1. Statistical analysis between coupled and uncoupled tissues, for the different conditions. No significant differences were found between control, K-12 and ΔetPetK comparing the coupled and uncoupled conditions.

iroB F	GCGTTAACGGTCATGAAG
iroB R	GGTAACCAGCTTCGGAAT
iroC F	CGGCACAGTCATGGATAG
iroB R	CAGCCAGCAAAATAGTCG

Table S2. Primers for detection of *iroB* and *iroC*
